# *AKT1*^E17K^ mutation profiling in breast cancer: prevalence, concurrent oncogenic alterations, and blood-based detection

**DOI:** 10.1186/s12885-016-2626-1

**Published:** 2016-08-11

**Authors:** Marion Rudolph, Tobias Anzeneder, Anke Schulz, Georg Beckmann, Annette T. Byrne, Michael Jeffers, Carol Pena, Oliver Politz, Karl Köchert, Richardus Vonk, Joachim Reischl

**Affiliations:** 1 Bayer Pharma AG, Muellerstrasse 178, 13353 Berlin, Germany; 2 Patients’ Tumor Bank of Hope (PATH), Augsburg, Germany; 3 Department of Physiology and Medical Physics, Royal College of Surgeons in Ireland, Dublin, Ireland; 4 Bayer HealthCare Pharmaceuticals, Whippany, NJ USA; 5 AstraZeneca R&D, Personalized Healthcare and Biomarkers, Gothenburg, Sweden; 6 At the time of manuscript preparation, the author was on a Science Foundation Ireland-funded industry secondment, Bayer HealthCare Pharmaceuticals, Whippany, NJ USA

**Keywords:** Breast cancer, *AKT1*^E17K^ mutation, Blood-based mutation detection

## Abstract

**Background:**

The single hotspot mutation *AKT1* [G49A:E17K] has been described in several cancers, with the highest incidence observed in breast cancer. However, its precise role in disease etiology remains unknown.

**Methods:**

We analyzed more than 600 breast cancer tumor samples and circulating tumor DNA for *AKT1*^E17K^ and alterations in other cancer-associated genes using Beads, Emulsions, Amplification, and Magnetics digital polymerase chain reaction technology and targeted exome sequencing.

**Results:**

Overall *AKT1*^E17K^ mutation prevalence was 6.3 % and not correlated with age or menopausal stage. *AKT1*^E17K^ mutation frequency tended to be lower in patients with grade 3 disease (1.9 %) compared with those with grade 1 (11.1 %) or grade 2 (6 %) disease. In two cohorts of patients with advanced metastatic disease, 98.0 % (*n =* 50) and 97.1 % (*n =* 35) concordance was obtained between tissue and blood samples for the *AKT1*^E17K^ mutation, and mutation capture rates of 66.7 % (2/3) and 85.7 % (6/7) in blood versus tissue samples were observed. Although *AKT1*-mutant tumor specimens were often found to harbor concurrent alterations in other driver genes, a subset of specimens harboring *AKT1*^E17K^ as the only known driver alteration was also identified. Initial follow-up survival data suggest that *AKT1*^E17K^ could be associated with increased mortality. These findings warrant additional long-term follow-up.

**Conclusions:**

The data suggest that *AKT1*^E17K^ is the most likely disease driver in certain breast cancer patients. Blood-based mutation detection is achievable in advanced-stage disease. These findings underpin the need for a further enhanced-precision medicine paradigm in the treatment of breast cancer.

**Electronic supplementary material:**

The online version of this article (doi:10.1186/s12885-016-2626-1) contains supplementary material, which is available to authorized users.

## Background

Metastatic breast cancer is a major cause of global cancer mortality and, despite several advances in recent years, is still largely incurable [1]. Critically, little progress has been made in the past decade in the evolution of chemotherapeutic or endocrine therapies to improve overall survival in patients. Nevertheless, targeted therapies, such as those directed against tumors overexpressing human epidermal growth factor receptor 2 (HER2), have improved patient outcomes [2]. Moreover, molecular-characterization studies in breast cancer have revealed that, in addition to HER2 amplification, tumors may possess numerous other genomic alterations located in oncogenes or tumor suppressor genes [3, 4]. As specific oncogenic events may be blocked by targeted therapies, screening for targetable genomic alterations may help to identify subpopulations of patients for whom specific targeted therapy would be beneficial.

One such targetable alteration resides in the v-akt murine thymoma viral oncogene (*AKT*). *AKT1* is a member of the serine-threonine kinase class that plays a key role in cellular processes, including growth, proliferation, survival, and angiogenesis. It is a downstream mediator of phosphatidylinositol 3-kinase which, along with *AKT1*, is a key mediator of proliferation and survival pathways frequently activated in cancer [5–10]. Tumors from patients with breast, colorectal, ovarian, and leukemic cancers have been shown to harbor activating somatic mutations in *AKT1* [5, 9]. The activation of *AKT1* is driven by membrane localization which, in turn, is initiated by the binding of the pleckstrin homology domain to phosphatidylinositol-3,4,5-trisphosphate or phosphatidylinositol-3,4-bisphosphate, followed by phosphorylation of the regulatory amino acids serine 473 and threonine 308 [7, 11].

Genetic mutations in the *AKT* pleckstrin homology domain have been reported to disturb the localization behavior and loss of sensitivity towards phosphatidylinositols, and to have major consequences in *AKT* functional behavior [5]. For instance, a somatic point mutation at nucleotide 49 introduces a lysine substitution for glutamic acid at amino acid 17 (*AKT1*^E17K^), resulting in a pathologic association of *AKT1* with the plasma membrane and constitutive activation of the enzyme which, in turn, results in an increased level of *AKT* phosphorylation and downstream molecules independent of upstream, e.g. stimulation of growth factor.

For breast cancer patients, *AKT1*^E17K^ mutation frequencies between 1.4 % and 8.2 %, with a mean mutation frequency of 3.8 %, have been described [12]. Moreover, the *AKT1*^E17K^ mutation appears to be restricted to ductal and lobular histotypes, and hormone receptor (HR)-positive breast tumors [13–15]. Interestingly, higher incidences of *AKT1*^E17K^ mutations have been reported to occur in benign papillomas (33 %; 20/61 [defined as papillomas without atypia]), compared with papillary carcinoma (10 %; 1/10) [16].

Several studies have indicated *PTEN*, *PIK3CA*, and *AKT1* mutations to be mutually exclusive (i.e. not co-occurring in the same tumor tissue sample) in individual tumors [5, 13, 17], suggesting that mutational activation of the phosphatidylinositol 3-kinase pathway by any one of these means is biologically equivalent. Alterations in all three are considered to be potential drivers of human breast cancer [4, 18]. However, particularly for *AKT1*^E17K^ mutation, the precise role in cancer development and progression in the clinical context is still largely unknown.

To better understand the role of the *AKT1*^E17K^ mutation in breast cancer, more than 600 tumor samples from breast cancer patients were profiled for presence of the *AKT1*^E17K^ mutation using Beads, Emulsions, Amplification, and Magnetics technology (BEAMing; Sysmex Inostics GmbH, Hamburg, Germany) in tissue and circulating tumor DNA (ctDNA). Additionally, targeted exome sequencing was conducted on tumor tissues to reveal any co-existence of the *AKT1*^E17K^ mutation with other oncogenic alterations.

## Methods

### Clinical samples and ethics

**Fig. 1:**
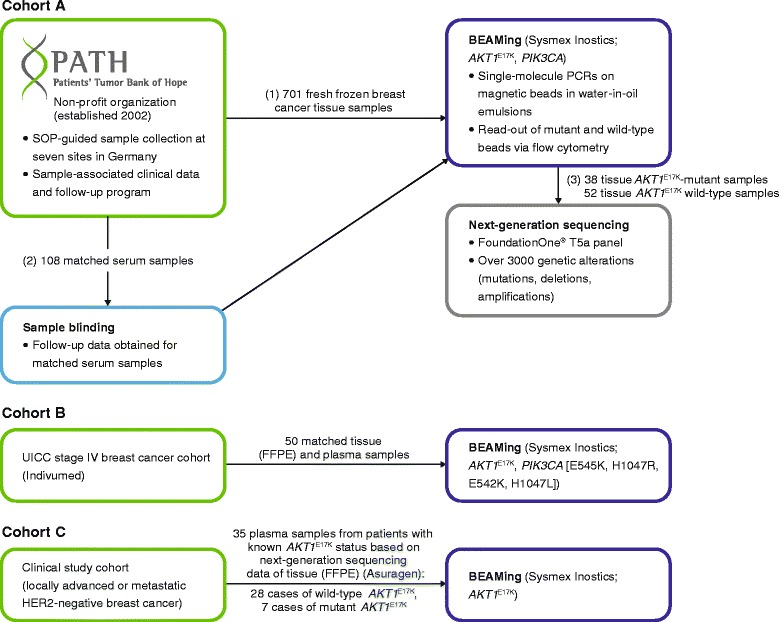
Sample flow and analysis. (1) 701 samples of breast cancer tumor tissue were obtained from PATH Biobank and analyzed with BEAMing for *AKT1* ^E17K^. (2) For a sub-cohort (108) of BEAMing-identified *AKT1* ^E17K^-mutant and wild-type samples, follow-up data were collected and matched serum was ordered. Serum samples were analyzed in a blinded fashion for *AKT1* ^E17K^ (BEAMing). (3) BEAMing-identified *AKT1* ^E17K^-mutant samples and a sub-cohort of wild-type samples were analyzed by next-generation sequencing. *Abbreviations: AKT1* v-akt murine thymoma viral oncogene, *BEAMing* Beads, Emulsions, Amplification, and Magnetics, *FFPE* formalin-fixed paraffin-embedded, *HER2* human epidermal growth factor receptor 2, PATH, Patients’ Tumor Bank of Hope, *PCR* polymerase chain reaction, *SOP* standard operating procedure, *UICC* Union for International Cancer Control

Samples were provided by the non-profit organization Patients’ Tumor Bank of Hope (PATH Biobank, Augsburg, Germany: http://www.path-biobank.org/index.php/en/about-path/) [19] as standardized fresh frozen tissue and blood serum specimens (cohort A; Fig. 1). Patients provided written, informed consent for the storage of samples and data, follow-up contact, and further use of samples and data for research purposes. The processes described were approved by the Bavarian Data Protection Commissioner and the ethics committee of the University of Bonn. Union for International Cancer Control (UICC) stage I–IV samples were selected based on: follow-up being possible; no previous treatment; primary disease; and estrogen receptor (ER)-positive status. The majority of serum samples (UICC stages I–III) were frozen within 3 h. Samples from neoadjuvantly treated patients were also ER-positive, but for relapsed patients both ER-positive and ER-negative samples were accepted.

A further cohort of paired, concurrently collected breast cancer tumor samples (formalin-fixed paraffin-embedded) and blood samples (plasma) from 50 patients with UICC stage IV disease were obtained by Indivumed GmbH (Hamburg, Germany) (cohort B; Fig. 1). In 45 of these cases, the tumor specimen collected was the primary breast tumor; the remaining five tissue samples were from biopsies of a metastatic breast cancer lesion. Samples were collected ethically within the framework of the “Hamburger Krankenhausgesetz 12a”.

A third cohort (cohort C; Fig. 1) comprised formalin-fixed paraffin-embedded tumor and plasma samples from patients with locally advanced or metastatic HER2-negative breast cancer enrolled in a clinical trial. The respective study protocol was approved by the institutional review board of each participating institution and complied with the Declaration of Helsinki, existing Good Clinical Practice guidelines, and local laws and regulations. All participants provided written, informed consent before enrollment.

### Tumor specimens and analysis workflow

Figure 1 depicts the overall sample flow and analysis plan for the study. In cohort A, 701 breast cancer samples were obtained from PATH Biobank. Specimens from untreated, ER-positive breast cancer patients (UICC stages I–IV) and ER-positive, neoadjuvantly treated patients as well as of relapsed ER-positive and ER-negative patient were collected and analyzed by BEAMing. Follow-up data were collected for the *AKT1*^E17K^ mutant samples and a closely matched subset of wild-type samples, based on clinical parameters e.g. disease stage and age (see in Additional file 1: Table S1). For the subgroup with UICC IV disease wild-type samples were selected randomly as no *AKT1*^E17K^ mutant sample was found in this patient group. Matching blood samples (serum) were ordered for these mutant and wild-type samples. In addition, tissue samples of all *AKT1*^E17K^ mutant samples and a subset of wild-type samples (see in Additional file 1: Table S2) were further analyzed by targeted exome sequencing (FoundationOne®, Cambridge, MA, USA) as described below.

Additionally, paired tissue and blood samples (plasma) from cohort B (*n =* 50) and blood samples (plasma) from cohort C (*n =* 35) with known *AKT1*^E17K^ status (determined by next-generation sequencing analysis [Asuragen, Inc., Austin, TX, USA] based on tissue) were analyzed by BEAMing.

### BEAMing

Analysis of tumor tissue and blood samples was performed by Sysmex Inostics. One to three tissue sections were scraped from glass slides and the entire sample was used for subsequent isolation of DNA, according to the manufacturer’s instructions (Epicentre, Madison, WI, USA). Blood samples were thawed at room temperature for approximately 15–30 min prior to DNA preparation. Cell debris was pelleted by centrifugation, and the supernatant was digested with proteinase K and purified according to the QIAamp DNA purification kit (QIAGEN GmbH, Hilden, Germany). Primers were designed to amplify a 96 bp region within the abundant consensus region of the human LINE-1 family. Quantitative real-time polymerase chain reaction (PCR) was performed in the presence of SYBR® Green I dye (Molecular Probes®, Inc., Eugene, OR, USA). An aliquot of the blood DNA was used as a template for the quantitative real-time PCR. Dilutions of normal human genomic DNA were run in parallel on each plate to serve as reference standards for the quantification of genomic DNA. Each sample and reference standard was run in duplicate. The threshold cycle number was determined using Eppendorf analysis software (Eppendorf AG, Hamburg, Germany) with PCR baseline subtracted. In a first pre-amplification step, multiple loci were amplified in a multiplex PCR reaction. In a second amplification step, nested primers were used for the amplification of individual amplicons. PCR products were quality-checked on agarose gel. Pre-amplified DNA was used for the subsequent BEAMing assay. Normalization was based on the Invitrogen Quant-iT™ PicoGreen® dsDNA reagent (Life Technologies, Carlsbad, CA, USA). The DNA content of PCR products was quantified by the automated liquid-handling system from Beckman Coulter, Inc. (Brea, CA, USA) connected to a fluorescence microplate reader. After the quantification step, samples were diluted in order to obtain a specific amount of pre-amplified DNA. Emulsion PCR enables the amplification of pre-amplified DNA fragments on the surface of magnetic beads that proceed in water-in-oil emulsions. Emulsions were subjected to standard thermal cycling conditions. Subsequently, the uncovered DNA fragments on the bead surface were hybridized using fluorescently labeled probes specific to the mutations of interest. The fluorescently labeled beads were quantified using flow cytometry. For the analysis of each base change, a separate flow cytometry analysis was performed. The result of a BEAMing assay is the fraction of mutant DNA alleles to wild-type DNA alleles present in a particular sample. This fraction is calculated by dividing the number of mutant beads by the total number of beads with PCR product (equal to the sum of mutant beads, mixed beads, and wild-type beads). The sensitivity for the *AKT1* mutation 49 G > A (E17K) is 0.02 % in blood and 1 % in tissue. The sensitivity is dependent on the presence of sufficient DNA molecules in the sample.

### Targeted sequencing: Foundation Medicine solid-tumor assay

The Foundation Medicine solid-tumor assay (FoundationOne® T5a panel) is a validated next-generation sequencing-based cancer genome profiling test that interrogates 4557 exons of 287 cancer-related genes with established performance benchmarks supporting direct clinical use [20]. Briefly, DNA was extracted from 90 tissue samples received from PATH Biobank (Fig. 1), 50–200 ng of which underwent whole-genome shotgun library construction and hybridization-based capture of 4557 exons from 287 cancer-related genes and 47 introns from 19 genes frequently rearranged in solid tumors. Using the HiSeq 2000 platform (Illumina, Inc., San Diego, CA, USA), hybrid-capture-selected libraries were sequenced to high uniform depth (targeting over 500× coverage by non-PCR duplicate read pairs, with over 99 % of exons at coverage over 100×). Sequence data were processed using a customized analysis pipeline designed to accurately detect multiple classes of genomic alterations (base substitutions, indels, focal gene amplifications, homozygous gene deletions, and selected gene fusions) in routine clinical specimens. Matched normal specimens were not analyzed; however, all reported mutations or classes of mutations have been identified in previously published cancer-sequencing studies and were therefore considered likely drivers of cancer. An alteration was categorized as “known” if reported as somatic in the COSMIC database (Wellcome Trust Sanger Institute, Genome Research Limited, Hinxton, Cambridge, UK). “Likely mutation” indicates a previously unknown, truncating mutation in a tumor suppressor, and “mutation of unknown impact” is a variant with unknown somatic/functional status. All testing was performed in a Clinical Laboratory Improvement Amendments-certified, College of American Pathologists-accredited laboratory.

### Statistical analyses

#### BEAMing and targeted sequencing

Likelihood-ratio tests were used to assess potential differences in *AKT1*^E17K^ mutation in breast cancer patients for the following clinical parameters: histologic subtypes (invasive ductal, invasive lobular, mixed, others); St. Gallen criteria [21]; grading; stage of disease (UICC stages I–IV); distal metastasis (M); lymph-node metastasis (yes/no); number of lymph-node metastases (categorical: 0, 1, 2, or 3); HER2 status comparing high HER2 expression (3+) analysis versus low (2+, 1+ or no expression) according to immunohistochemistry (IHC); menopausal stage (pre or post); age (ordinal); progesterone-receptor expression; and tumor size (T; based on the tumor node metastasis classification). In addition to the respective *P*-value, Bonferroni-Holm adjusted *P*-values were reported to account for multiple testing. The correlation between mutant allele frequencies, as detected independently by BEAMing and the Foundation Medicine solid-tumor assay, was assessed by calculating the Pearson product–moment correlation coefficient (Pearson’s *r*). Fisher’s exact test was employed to compare mutant versus wild-type *AKT1* samples for enrichment of one of the detected mutations in any of the 235 genes. Correction of the *P*-values for multiple testing was done by the Benjamini-Hochberg method [22].

#### Survival estimation

Survival time was calculated based on the time from the date of surgery until the date of death. For surviving patients, the date of follow-up was used as a censored observation. Analyses were performed using a Cox proportional hazards model, with *AKT1*^E17K^ mutation as the main factor, together with age at diagnosis and disease category (with levels: UICC I-IV, relapse, neoadjuvantly treated) as covariates. Kaplan-Meier estimates were displayed for *AKT1* wild-type and mutant status.

## Results

### *AKT1*^E17K^ prevalence in breast cancer subgroups

Of the 701 samples in cohort A, 619 samples were evaluable for the *AKT1*^E17K^ mutation: 79 from neoadjuvantly treated patients, 46 from relapsed patients, and 494 from untreated patients (Table 1). Samples of untreated, newly diagnosed patients were categorized according to conventional UICC staging: UICC stage I (T1, N0, M0); UICC stage II (T2, N0–1, M0, and T1, N1, M0); UICC stage III (any T, N2–3, M0, and T3 or T4, any N, M0); and UICC stage IV (any T, any N, M1). Eighty-two (11.7 %) samples had not sufficient tumor content according to pathologic examination, and thus could not be evaluated for *AKT1* mutations. The failure rate was distributed as follows between the different patient sample groups: 11.8 % (19/161) in samples from patients with UICC stage I disease; 6.6 % (14/212) in samples from patients with UICC stage II disease; 3.5 % (4/114) in samples from patients with UICC stage III disease; and 2.2 % (1/45) in samples from patients with UICC stage IV disease. As expected, the highest failure rate (33.6 %; 40/119) was observed in the neoadjuvantly treated patient group (data not shown).

**Table 1:** Prevalence of the *AKT1* ^E17K^ mutation in untreated (UICC stages I–IV), neoadjuvantly treated, or relapsed breast cancer patients

	Total *n*	*AKT1* ^E17K^ *n* (%)	Wild type *n* (%)	95 % CI
Neoadjuvant^a^	79	5 (6.3)	74 (93.7)	2.1–14.2
Relapsed^b^	46	5 (10.9)	41 (89.1)	3.6–23.6
UICC stages I–IV^a^	494	29 (5.9)	465 (94.1)	4.0–8.3
*Overall*	619	39 (6.3)	580 (93.7)	4.5–8.5

DNA was obtained from fresh frozen tumor tissue and analyzed using BEAMing

^a^ER-positive patients

^b^ER-positive and ER-negative patients

*Abbreviations: 95 % CI* 95 % exact confidence interval, *AKT1* v-akt murine thymoma viral oncogene, *ER* estrogen receptor, *UICC* Union for International Cancer Control

Overall, the prevalence of *AKT1*^E17K^ mutation in tumor samples was 6.3 % (39/619; 95 % confidence interval 4.5–8.5) (Table 1). The highest mutation frequency (10.9 %; 95 % confidence interval 3.6–23.6) was observed in samples from relapsed patients, although this represented a small number of samples (5/46). In previously untreated patients, an *AKT1*^E17K^ mutation was identified in 9.2 % (13/142), 5.6 % (11/198), and 4.5 % (5/110) of patients with UICC stage I, II, or III disease, respectively (Table 2). No patients with UICC stage IV disease (0/44) were shown to harbor the *AKT1*^E17K^ mutation.

**Table 2:** Clinical parameters and association with *AKT1* ^E17K^ mutation in previously untreated breast cancer patients (cohort A)

Parameter	Total (*N =* 494)	Mutation		*P*-value^a^ (adjusted *P*-value)
		Wild type (*n =* 465)	*AKT1* ^E17K^ (*n =* 29)	
Age, years				0.55 (1.00)
< 35	8 (1.6)	8 (1.7)	0	
35–65	255 (51.6)	241 (51.8)	14 (48.3)	
> 65	231 (46.8)	216 (46.5)	15 (51.7)	
Menopausal status^b^				0.34 (1.00)
Pre	77 (15.6)	74 (15.9)	3 (10.3)	
Post	381 (77.1)	356 (76.6)	25 (86.2)	
UICC stage				0.04 (0.47)
I	142 (28.7)	129 (27.7)	13 (44.8)	
II	198 (40.1)	187 (40.2)	11 (37.9)	
III	110 (22.3)	105 (22.6)	5 (17.2)	
IV	44 (8.9)	44 (9.5)	0	
Grade				0.03 (0.38)
1	72 (14.6)	64 (13.8)	8 (27.6)	
2	315 (63.8)	296 (63.7)	19 (65.5)	
3	107 (21.7)	105 (22.6)	2 (6.9)	
Lymph-node metastasis (N stage)^c^				0.17 (1.00)
N0	238 (48.2)	223 (48.0)	15 (51.7)	
N1	138 (27.9)	128 (27.5)	10 (34.5)	
N2	78 (15.8)	77 (16.6)	1 (3.4)	
N3	39 (7.9)	36 (7.7)	3 (10.3)	
Distant metastasis (M stage)				0.02 (0.25)
M0	450 (91.1)	421 (90.5)	29 (100)	
M1	44 (8.9)	44 (9.5)	0	
Histology^d^				0.83 (1.00)
Ductal	368 (74.5)	346 (74.4)	22 (75.9)	
Lobular	89 (18.0)	85 (18.3)	4 (13.8)	
Mixed	10 (2.0)	9 (1.9)	1 (3.4)	
Others	23 (4.7)	21 (4.5)	2 (6.9)	
St. Gallen criteria^e^				0.19 (1.00)
Luminal A	210 (42.5)	194 (41.7)	16 (55.2)	
Luminal B1	53 (10.7)	52 (11.2)	1 (3.4)	
Luminal B2	231 (46.8)	219 (47.1)	12 (41.4)	
HER2 status				0.03 (0.38)
IHC-Score (3+)	37 (7.5)	37 (8.0)	0	
IHC-Score (0 - 2+)	457 (92.5)	428 (92.0)	29 (100)	
PR status^f^				0.91 (1.00)
Negative	43 (8.7)	41 (8.8)	2 (6.9)	
Positive	375 (75.9)	356 (76.6)	19 (65.5)	

^a^Likelihood-ratio test for the hypothesis that the prevalence of *AKT1* ^E17K^ mutation in breast cancer patients does not differ based on the respective clinical parameter. Displayed are only selected parameters

^b^Missing, *n =* 36. ^c^Missing, *n =* 1. ^d^Missing, *n =* 4. ^e^tumor grade was used instead of Ki-67 for subgrouping. ^f^Missing, *n =* 76

*Abbreviations: AKT1* v-akt murine thymoma viral oncogene, *HER2* human epidermal growth factor receptor 2, IHC immunohistochemistry, *PR* progesterone receptor, *UICC* Union for International Cancer Control

### Association of *AKT1*^E17K^ with clinical parameters

No association of *AKT1*^E17K^ mutation prevalence was found with respect to age, menopausal stage, histologic subtype, lymph-node metastasis (N stage), progesterone-receptor status, or St. Gallen criteria (applying the definition by Brouckaert et al., whereby tumor grade replaces Ki-67) [21] considering untreated breast cancer patients from cohort A (Table 2). However, the latter finding might reflect that this patient cohort was ER-positive. *AKT1*^E17K^ mutations appeared to be associated with lower HER2 expression. 6.3 % (29/457) of patients with an IHC score of 0, 1+ or 2+ harbored a mutation, compared with 0 % (0/37) of HER2 3+ patients.

No patients (untreated breast cancer, cohort A) with UICC stage IV disease harbored the *AKT1*^E17K^ mutation. Surprisingly, patients with poorly differentiated tumors (grade 3) had the lowest prevalence of *AKT1*^E17K^ mutation (1.9 %; 2/107), whereas patients with well-differentiated tumors (grade 1) and moderately differentiated tumors (grade 2) exhibited a prevalence of *AKT1*^E17K^ mutation of 11.1 % (8/72) and 6 % (19/315), respectively (Table 2). Of note, *AKT1*^E17K^ mutations were observed in patients with invasive ductal phenotype (6.0 %; 22/368), lobular disease (4.7 %; 4/89), mixed histotype (10 %; 1/10), or other (8.7 %; 2/23; papillary and ductal, tubular carcinoma).

### Association of *AKT1*^E17K^ with survival

**Fig. 2:**
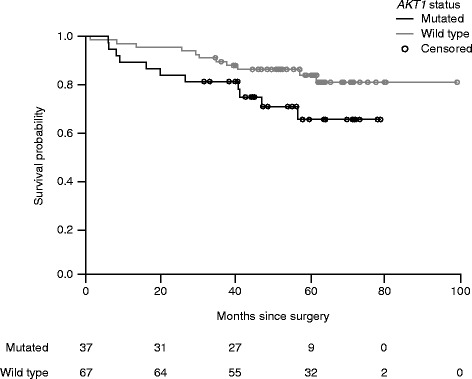
Survival of patients with mutant *AKT1* ^E17K^ (*n =* 37) or wild-type *AKT1* ^E17K^ (*n =* 67). Survival was calculated from the date of surgery. Date of death was taken if available; for surviving patients, the date of follow-up was taken and censored. Age and disease category adjusted hazard ratio was 0.232, with a 95 % confidence interval ranging from 0.071 to 0.754 (*P* = 0.015). Circles denote censored observations. Number of subjects at risk are given. *Abbreviation: AKT1* v-akt murine thymoma viral oncogene

Survival information was available for 104 patients. The median follow-up time was 55.3 months, ranging from 1.13 months to the first death, to 99.2 months (survival). Of these 104 patients, 22 died from any cause (21.2 %; link of mortality to malignancy unknown), and 82 patients survived until follow-up. Wild-type *AKT1* patients had a slightly lower mortality rate (16.4 %; 11/67) than those presenting with mutant *AKT1* (29.7 %; 11/37) (Fig. 2). The age and disease category adjusted hazard ratio was 0.232; 95 % confidence interval 0.0710.754 (*P* = 0.015). For patients with UICC stage I–III disease, deaths were reported for approximately 2 % (1/42) for the *AKT1*^E17K^ wild-type group, compared with approximately 22 % (6/27) for the mutant group. Recurrence-free survival could not be determined as these data were only partially available.

### Detection of *AKT1*^E17K^ and *PIK3CA* mutations in matched blood samples

As the interrogation of blood-based clinical-mutation detection plays an increasingly important role, we assessed whether *AKT1*^E17K^ mutations detected via BEAMing in tumor tissue could be found in the corresponding ctDNA. In paired tissue and blood samples from cohort A, 67.0 % concordance for *AKT1*^E17K^ mutation was found (Table 3). However, the majority of correctly matched samples were wild-type *AKT1*^E17K^. Detailed analyses of mismatched samples indicated that samples detected as mutant *AKT1*^E17K^ in tumor tissue were often not confirmed for mutational status in blood samples. Of 35 samples indicated as mutant *AKT1*^E17K^ using tumor tissue in cohort A, the mutational status was confirmed for four patients using ctDNA, representing an *AKT1*^E17K^ mutation capture rate of 11.4 % (4/35) for ctDNA. In patients with UICC stage IV breast cancer, 98.0 % (cohort B) and 97.1 % (cohort C) concordance rates were observed for the *AKT1*^E17K^ mutation between paired tumor and blood samples. In these cohorts, *AKT1*^E17K^ mutations identified in tumor tissue could be confirmed in blood in two out of three patients (cohort B; capture rate of 66.7 % for ctDNA) and in six out of seven patients (cohort C; capture rate of 85.7 % for ctDNA). Interestingly, tissue was collected for two of these patients more than 3 years before the blood sample was collected.

**Table 3:** Comparative mutation analysis using blood and tissue samples of the same patients

			*AKT1* ^E17K^			*PIK3CA*	
			Cohort A	Cohort B	Cohort C	Cohort A	Cohort B
Total paired samples analyzed, *n*			94	50	35	71	50
Mismatched samples, pairs, *n*							
	Tumor	Blood				-	-
	*AKT1* WT	*AKT1* ^E17K^	0	0	0	**-**	**-**
	*AKT1* ^E17K^	*AKT1* WT	31	1	1	**-**	**-**
	*PIK3CA* WT	*PIK3CA* mut	**-**	**-**	**-**	1	0
	*PIK3CA* mut	*PIK3CA* WT	**-**	**-**	**-**	16	0
	*PIK3CA* mut	*PIK3CA* mut	**-**	**-**	**-**	1	0
Total mismatched, pairs, *n*			31	1	1	18	0
Matched samples, pairs, *n*							
	*AKT1* WT	*AKT1* WT	59	47	28	**-**	**-**
	*AKT1* ^E17K^	*AKT1* ^E17K^	4	2	6	**-**	**-**
	*PIK3CA* WT	*PIK3CA* WT	**-**	**-**	**-**	50	37
	*PIK3CA* mut	*PIK3CA* mut	**-**	**-**	**-**	5	13
Total matched, pairs, *n*			63	49	34	55	50
Concordance (%)			67.0	98.0	97.1	75.3^a^	100
Mutation capture rate in ctDNA (%)			11.4	66.7	85.7	22.7	100

Cohort A: Analysis of *AKT1* ^E17K^ mutations using BEAMing for fresh frozen tumor tissue samples and blood samples (serum), and analysis of *PIK3CA* mutations (H1047R, H1047L, E542K, E545K) using BEAMing for blood samples (serum) and next-generation sequencing for fresh frozen tumor tissue samples. Cohort B: Both *AKT1* ^E17K^ and *PIK3CA* mutations (H1047R, H1047L, E542K, E545K) were determined in tissue (FFPE) and blood (plasma) using BEAMing. Cohort C: Analysis of *AKT1* ^E17K^ using BEAMing for blood samples (plasma) and next-generation sequencing for tissue samples (FFPE). “Matched samples” indicates that the same result was obtained using tissue or blood samples. “Mismatched samples” indicates that different results were obtained using tissue and blood samples. ^a^Calculation was based on total measured events (*n =* 73), as in one sample, two *PIK3CA* mutations were detected of which only one matched

*Abbreviations: AKT1* v-akt murine thymoma viral oncogene, *BEAMing* Beads, Emulsions, Amplification, and Magnetics, *FFPE* formalin-fixed paraffin-embedded, *mut* mutant, *WT* wild type

*PIK3CA* mutations (H1047R, H1047L, E542K, E545K) were analyzed by BEAMing in the same blood samples used for *AKT1*^E17K^ detection (Table 3). For cohort A, *PIK3CA* mutation profiling in tissue was obtained by next-generation sequencing. Comparable with the results for *AKT1*^E17K^, there was 75.3 % concordance between the results obtained from tissue and blood, and a mutation capture rate of 22.7 % for ctDNA was found for *PIK3CA* mutations in cohort A. Non-matching results were mainly based on the ability to detect the mutation in tissue but not blood samples. Only in a single case was a mutation detected in blood but not in tumor; in another case, a different *PIK3CA* mutation was detected in tumor compared with blood. In patients with advanced breast cancer (cohort B), 100 % concordance, as well as a 100 % mutation capture rate for ctDNA versus tissue DNA, was observed for *PIK3CA* by BEAMing (Table 3).

### Co-existence of the *AKT1*^E17K^ mutation with oncogenic driver mutations and further genetic alterations

**Fig. 3:**
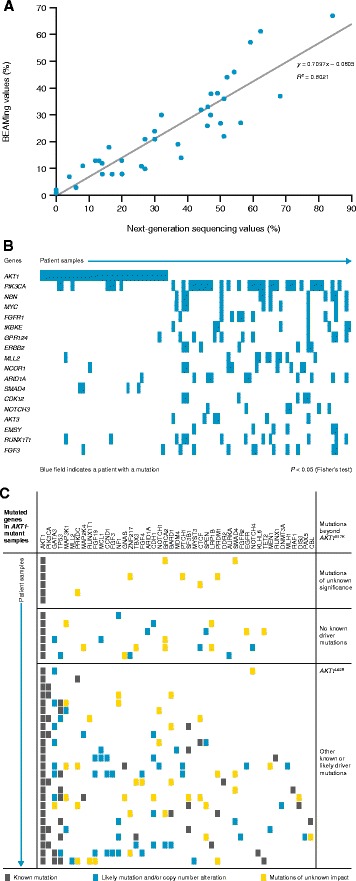
Characterization of *AKT1*-mutant and wild-type samples. **a** Mutant allele frequencies for *AKT1* ^E17K^ mutations as identified by next-generation sequencing and BEAMing (Pearson correlation coefficient = 0.9). **b** Comparison of mutant versus wild-type *AKT1* samples shows significantly different mutation spectra. Shown are mutations in genes that are distributed significantly differently between groups (*P* < 0.05, Fisher’s test, not corrected for multiple testing). Four of the seven *PIK3CA* mutations in mutant samples have low mutant allele frequencies (≤3 %). **c** *AKT1*-mutant samples as characterized by next-generation sequencing. All additionally mutated genes are shown that have known functional impact (gray box, known single nucleotide variants or indels) or likely functional impact (blue box, likely single nucleotide variants or copy number alterations or rearrangements). Yellow boxes indicate variants with unknown somatic/functional status in the listed genes with alterations of known or likely impact. Variants with unknown somatic/functional status found in additional genes of the FoundationOne® T5a panel are not depicted. *Abbreviations: AKT1* v-akt murine thymoma viral oncogene, *BEAMing* Beads, Emulsions, Amplification, and Magnetics

Comprehensive profiling of *AKT1*^E17K^ may help to define a potential role for the mutation in the development and progression of breast cancer. Thus, we selected the 38 cases identified by BEAMing bearing the *AKT1*^E17K^ mutation as well as 52 wild-type samples for targeted sequencing. Of these, one wild-type sample did not contain sufficient material for targeted sequencing. Mutant allele frequencies for *AKT1*^E17K^, as detected independently by BEAMing and FoundationOne® targeted sequencing, correlated well (R^2^ = 0.8021; Fig. 3a). Mutational status for just two of the 38 samples (identified by BEAMing as bearing the *AKT1*^E17K^ mutation) could not be reconfirmed by targeted sequencing. However, both cases had a rather low mutation frequency of 1 % and 2.3 %, respectively. A total of 235 cancer-related genes were affected by 1131 mutations (amplifications, deletions, mutations). In the *AKT1*-mutant cohort (*n =* 37; one sample with *AKT1*^L52R^), 158 genes were affected, with 213 genes affected in the non-*AKT1*-mutant cohort. Eighty-one genes were mutated in at least 5 % of samples, among them 13 from the top 20 list of most frequently mutated breast cancer genes in the COSMIC database (cancer.sanger.ac.uk) [23]: (*PIK3CA*, *AKT1*, *GATA3*, *TP53*, *MLL2*, *MAP2K4*, *NF1*, *ARID1A*, *CDH1*, *MED12*, *PTEN*, *BRCA1*, *APC*). A comparison of additional gene alterations associated with *AKT1*-mutant versus wild-type tumors identified some genes that were altered exclusively in *AKT1*-mutant (*SMAD4*) or *AKT1* wild-type (*CDK12*, *NOTCH3*, *AKT3*, *EMSY*, *ERBB2*, *NBN*, *MYC*, *FGFR1*, *IKBKE*) tumors (Fig. 3b). In addition, some genes were altered in both *AKT1*-mutant and wild-type tumors, but of these only the *PIK3CA* mutation was significantly more likely to be associated with *AKT1* wild-type tumors (false discovery rate <0.1, Fisher’s exact test, after correction for multiple testing by Benjamini-Hochberg). *PIK3CA* mutation frequencies in *AKT1* wild-type and mutant samples were 58 % and 19 %, respectively. Furthermore, four of the seven *PIK3CA* mutations in *AKT1*-mutant samples had low mutant allele frequencies (<3 %). It is hypothesized that *AKT1* is a driver mutation in breast cancer which could be supported by identification of patients bearing no other known cancer-causing mutations. In this context, we identified 12 patients who had no additional mutations known to drive cancer (Fig. 3c). While six of these patients had further alterations which likely contribute to cancer development (e.g. truncating mutation in tumor suppressor and/or copy number alterations), no cause other than *AKT1* could be identified for the remaining six cases.

## Discussion

The single hotspot mutation in the pleckstrin homology domain of the *AKT1* gene [G49A:E17K] has been described in human breast, colon, and ovarian cancers, with the highest incidence observed in breast cancer [5]. Including all recently published studies, the reported frequency of this mutation ranges from 1.4 % to 12.5 %, with a mean frequency of 3.1 % [4, 5, 9, 13, 14, 24–37] (see in Additional file 1: Table S3). However, the majority of these studies (14/19) had relatively small sample sizes (under 200) and were associated with broad confidence intervals. While meta-analysis can support the identification of actual mutation frequencies, drawing conclusions about any association of mutation frequency and clinical parameters in this way is challenging, as available clinical information varies widely across studies.

We assessed the prevalence of the *AKT1*^E17K^ mutation in a large breast cancer cohort (over 600 cases, cohort A). Our data indicate a prevalence of 6.3 % (39/619; 95 % confidence interval 4.5–8.54) using BEAMing technology on tumor tissue, which is in agreement with previous studies (see in Additional file 1: Table S3). Assessment of ctDNA by BEAMing has been shown to reliably facilitate analysis of a cancer patient’s mutational status [38, 39]. However, using matched liquid biopsy samples, approximately 6 % prevalence could not be confirmed in this cohort, and the mutation capture rate in ctDNA was only 11.4 % (4/35). We obtained similar results for *PIK3CA* mutation. In samples from patients with advanced metastatic breast cancer (cohorts B and C), we obtained 98.0 % and 97.1 % concordance and an *AKT1*^E17K^ mutation capture rate of 66.7 % and 85.7 % for ctDNA, and 100 % concordance and a *PIK3CA* mutation capture rate of 100 % (cohort B). This is in line with concordance for *AKT1*^E17K^ (100 % in three patients) observed by Perkins et al. in patients eligible for a phase I study [25]. Interestingly, tissue samples from two patients in our cohort were collected several years before blood samples, thereby providing the first hint that the *AKT1*^E17K^ mutation is stable during disease development.

Blood samples for cohort A were serum, compared with plasma used in cohorts B and C. Thus, differences in mutation detection based on sample type cannot be excluded. However, considering all data obtained so far, the low mutation capture rate for ctDNA within cohort A is likely based on the fact that the majority of patients had primary-diagnosed early-stage disease without previous treatment (approximately 55 % for UICC stages I and II, and 7 % for UICC stage IV). Thus, these data are in line with previous studies indicating a stage-dependent limitation for mutation detection by ctDNA profiling [40–43].

The *AKT1*^E17K^ prevalence determined in our study was not correlated with age or menopausal stage. In line with findings described by Stemke-Hale et al. [14], we did not find any tumors expressing HER2 at a level of IHC-Score 3+ also bearing the *AKT1*^E17K^ mutation (0/37). Interestingly, we identified one HR-negative patient as having mutated *AKT1*^E17K^ (1/10; 3/20 for HR-positive) within the group of relapsed cases. Thus, it is possible that *AKT1*^E17K^ mutation may not be restricted to HR-positive breast cancer [14]. However, any predominance in HR-positive breast cancer as described for *PIK3CA* cannot be excluded because of the low number of cases in our HR-negative cohort [26].

It has been described that *AKT1*^E17K^ mutation could not be found in medullary and mucinous histotypes and is restricted to ductal and lobular histotypes [13, 15]. In contrast to this, we found the mutation also in a relapsed patient with mucinous carcinoma (grade 1, ER/progesterone receptor unknown). Thus, our data support the assertion that large cohorts containing sufficient numbers in each clinical subgroup are required to reliably evaluate mutation association with histotype or other clinical parameters. Furthermore, in this cohort, prevalence of the *AKT1*^E17K^ mutation was lower in patients with grade 3 disease compared with those with grade 1 or grade 2 disease. Comparable data were obtained for *PIK3CA* mutations in breast cancer patients [26].

This leads to the question as to why *AKT1*^E17K^ prevalence is reduced in the more advanced disease setting. It has been hypothesized that *AKT* activation confers a selective advantage during early HR-positive tumorigenesis but inhibits tumor dissemination during progression [14]. This is supported by the observation that over-activation of *AKT* drives initiation of tumorigenesis but inhibits invasion and metastasis in an ERBB-2-induced mammary tumor model [44]. Accordingly, *AKT1*^E17K^-mutant clones could play a role during early tumorigenesis and – contradictory to the assumption of clonal stability – be overgrown during disease progression from grade 1 to 3 by other clones.

On the other hand, assuming that tumors bearing the *AKT1*^E17K^ mutation are rather more aggressive and rapidly growing, one could hypothesize that patients bearing this tumor phenotype could present earlier and hence be diagnosed at an early disease stage (e.g. UICC stage I/grade 1), as inferred in the present work. Consequently, patients bearing the *AKT1*^E17K^ mutation would already be undergoing therapy when their disease reached UICC stage IV/grade 3 and would therefore not be identifiable in a first-diagnosis cohort as used herein. However, they would be identifiable in a cohort not limited to first-diagnosis patients corresponding to our cohort B (3 *AKT1*^E17K^-mutated patients out of 50 according to tissue analysis) or relapsed patients (5/45). Compared with patients with wild-type *AKT1*, patients with *AKT1*^E17K^ mutations would also therefore be expected to relapse sooner and/or sustain disease-related death. Long-term follow-up data from patients are required in order to confirm this hypothesis.

Although low sample numbers precluded comprehensive analysis, initial follow-up survival data indicate increased death rates in early-stage disease (UICC stages I–III) for patients with mutant *AKT1*^E17K^ versus wild type. Thus, *AKT1*^E17K^ could be a negative prognostic factor. However, in contrast to this, it has been reported that disease did not recur in six *AKT1*^E17K^-mutant patients [14]. Further collection of follow-up data in collaboration with PATH Biobank is planned. This will facilitate confirmation of the impact of *AKT1*^E17K^ (and also other mutations measured with the FoundationOne® panel) on disease progression or survival.

It has been described that *PIK3CA* and *AKT1* mutations are not co-occurring in individual tumors [5]. However, the sample size was insufficient to document statistical significance, and an exception (based on one case only) has been noted in another study where the patient bore both *PIK3CA* and *AKT1* mutations [13]. Our data clearly indicate that both mutations can co-exist within one tumor. However, *PIK3CA* mutations are rather under-represented in *AKT1*^E17K^-mutant compared with wild-type samples. Moreover, in *AKT1*-mutant samples, the *PIK3CA* allele frequencies were lower than the *AKT1*^E17K^ allele frequency. Thus, it is possible that the *AKT1*^E17K^ mutation occurred earlier in development in these specific cancer samples. Of course, since our analysis was performed on the entire tumor tissue, the possibility that *AKT1* and *PIK3CA* mutations existed in different cells within the tumor could not be excluded.

*AKT1*^E17K^ may represent a *bona fide* oncogene in the context of human luminal breast cancer. Recently, knock-in of the mutation into a luminal breast cancer cell-line model against a *PIK3CA* wild-type background was shown to restore pathway signaling, proliferation, and tumor growth *in vivo* [18]. Our data support the oncogenic role of *AKT1*^E17K^, as 16.2 % (6/37) of all *AKT1*-mutant patients were identified as having no other mutation or genetic alterations known to drive their disease or which were likely involved in disease generation or progression. Thus, *AKT1*^E17K^ in these patients was the most likely disease driver. To date, there are several clinical programs in place addressing *AKT* as a potential therapeutic target [45–47]. ATP-competitive inhibitors and allosteric inhibitors have shown promising efficacy in *AKT1*-mutant cancers in preclinical studies [48].

## Conclusions

Our data suggest that conclusions regarding the prevalence of rare mutations such as *AKT1*^E17K^ may only be drawn using large cohorts with associated well-documented clinical data. Integrative combined analyses of data emerging from such studies coupled with appropriate follow-up programs may support the interrogation of mutation phenotype, incidence, and association with disease initiation or progression into treatment decisions. Thus, outcomes from these analyses may ultimately be employed in the design of appropriate clinical trials towards an enhanced-precision medicine paradigm in breast cancer.

## Abbreviations

*AKT1,* v-akt murine thymoma viral oncogene; *BEAMing,* Beads, Emulsions, Amplification, and Magnetics; *ctDNA,* circulating tumor DNA; *ER,* estrogen receptor; *HER2,* human epidermal growth factor receptor 2; *HR,* hormone receptor; *IHC,* immunohistochemistry; *PATH,* Patients’ Tumor Bank of Hope; *PCR,* polymerase chain reaction; *UICC,* Union for International Cancer Control

## Additional files

Additional file 1: Table S1. Clinical parameters of patient samples for which survival data were collected. **Table S2.** Clinical parameters of patient samples for which FoundationOne® targeted sequencing was performed. **Table S3.** Published studies evaluating the prevalence of *AKT1* mutations in breast cancer patients. (DOCX 31 kb)
