# Supplementary material for: AKT1E17K mutation profiling in breast cancer: prevalence, concurrent oncogenic alterations, and blood-based detection
Source: BMC Cancer. 2016 Aug 11;16:622. doi: 10.1186/s12885-016-2626-1 (PMC4982009; doi:10.1186/s12885-016-2626-1)
Supplement: Additional file 1: Table S1. — Clinical parameters of patient samples for which survival data were collected. Table S2. Clinical parameters of patient samples for which FoundationOne® targeted sequencing was performed. Table S3. Published studies evaluating the prevalence of AKT1 mutations in breast cancer patients. (DOCX 31 kb) [file 12885_2016_2626_MOESM1_ESM.docx]

**Table S1.** Clinical parameters of patient samples for which survival data were collected

| **Parameter** | **Mutation status** | |
| --- | --- | --- |
|  | **Wild type  (n = 67)** | ***AKT1*^E17K^  (n = 37)** |
| **Neoadjuvant** | 6 | 5 |
| **Relapsed** | 7 | 5 |
| **UICC stage** |  |  |
| I | 17 | 12 |
| II | 17 | 10 |
| III | 8 | 5 |
| IV | 12 | 0 |
| **Age, years** |  |  |
| <35 | 1 | 0 |
| 35–65 | 33 | 18 |
| >65 | 33 | 19 |
| **Menopausal status** |  |  |
| Pre | 13 | 4 |
| Post | 54 | 33 |
| **Grade** |  |  |
| n/a^a^ | 2 | 3 |
| 1 | 12 | 9 |
| 2 | 34 | 21 |
| 3 | 19 | 4 |
| **Lymph-node metastasis (N stage)** |  |  |
| n/a | 4 | 2 |
| N0 | 30 | 18 |
| N1 | 20 | 10 |
| N2 | 5 | 2 |
| N3 | 8 | 5 |
| **Histology** |  |  |
| n/a | 1 | 0 |
| Ductal | 56 | 28 |
| Lobular | 4 | 5 |
| Mixed | 1 | 1 |
| Other | 5 | 3 |
| **HER2 status** |  |  |
| IHC-Score (3+) | 4 | 0 |
| IHC-Score (0–2+) | 63 | 37 |
| **PR status** |  |  |
| n/a | 11 | 8 |
| Negative | 9 | 6 |
| Positive | 47 | 23 |

^a^For neoadjuvantly treated patients grading of the tumor prior to chemotherapy may not be available. *Abbreviations: AKT1* v-akt murine thymoma viral oncogene, *HER2* human epidermal growth factor receptor 2, *IHC* immunohistochemistry, *PR* progesterone receptor, *UICC* Union for International Cancer Control

**Table S2.** Clinical parameters of patient samples for which FoundationOne^®^ targeted sequencing was performed

| **Parameter** | **Mutation** | |
| --- | --- | --- |
|  | **Wild type  (n = 51)** | ***AKT1*^E17K^  (n = 38)** |
| **Neoadjuvant** | 7 | 5 |
| **Relapsed** | 7 | 4 |
| **UICC stage** |  |  |
| I | 12 | 13 |
| II | 11 | 11 |
| III | 7 | 5 |
| IV | 7 | 0 |
| **Age, years** |  |  |
| <35 | 0 | 0 |
| 35–65 | 27 | 19 |
| >65 | 24 | 19 |
| **Menopausal status** |  |  |
| n/a | 0 | 1 |
| Pre | 7 | 4 |
| Post | 44 | 33 |
| **Grade** |  |  |
| n/a^a^ | 4 | 3 |
| 1 | 8 | 8 |
| 2 | 22 | 22 |
| 3 | 17 | 5 |
| **Lymph-node metastasis (N stage)** |  |  |
| n/a | 3 | 2 |
| N0 | 24 | 18 |
| N1 | 15 | 11 |
| N2 | 3 | 2 |
| N3 | 6 | 5 |
| **Histology** |  |  |
| n/a | 1 | 0 |
| Ductal | 45 | 30 |
| Lobular | 2 | 5 |
| Mixed | 0 | 1 |
| Other | 3 | 2 |
| **HER2 status** |  |  |
| IHC-Score (3+) | 3 | 0 |
| IHC-Score (0–2+) | 48 | 38 |
| **PR status** |  |  |
| n/a | 9 | 8 |
| Negative | 8 | 6 |
| Positive | 34 | 24 |

^a^For neoadjuvantly treated patients grading of the tumor prior to chemotherapy may not be available. *Abbreviations: AKT1* v-akt murine thymoma viral oncogene, *HER2* human epidermal growth factor receptor 2, *IHC* immunohistochemistry, *PR* progesterone receptor, *UICC* Union for International Cancer Control

**Table S3.** Published studies evaluating the prevalence of *AKT1* mutations in breast cancer patients

| **Author** | **N** | ***AKT1*^E17K^, n** | ***AKT1*^E17K^, %** | **Citation** |
| --- | --- | --- | --- | --- |
| Carpten | 60 | 5 | 8.3 | Nature 2007;448:439–44 |
| Kim | 78 | 4 | 5.1 | Br J Cancer 2008;98:1533–5 |
| Bleeker | 273 | 16 | 5.9 | Oncogene 2008;27:5648–50 |
| Stemke-Hale | 418 | 6 | 1.4 | Cancer Res 2008;68:6084–91 |
| Dunlap | 78 | 3 | 3.8 | Breast Cancer Res Treat 2010;120:409–18 |
| Kadota | 150 | 6 | 4.0 | Cancer Res 2009;69:7357–65 |
| Lauring | 100 | 3 | 3.0 | Oncogene 2010;29:2337–45 |
| Kan | 174 | 3 | 1.7 | Nature 2010;466:869–73 |
| Boyault | 120 | 5 | 4.2 | Breast Cancer Res Treat 2012;132:29–39 |
| Troxell | 8 | 1 | 12.5 | Mod Pathol 2012;25:930–7 |
| Santarpia | 267 | 5 | 1.9 | Breast Cancer Res Treat 2012;134:333–43 |
| Stephens | 100 | 4 | 4.0 | Nature 2012;486:400–4 |
| Arnedos | 108 | 6 | 5.6 | Eur J Cancer 2012;48:2293–9 |
| Parikh | 51 | 1 | 2.0 | Proc Natl Acad Sci U S A 2012;109:19368–73 |
| Perkins | 19 | 1 | 5.3 | PLoS One 2012;7:e47020 |
| Flatley | 19 | 1 | 5.3 | Hum Pathol 2013;44:1320–7 |
| Ross | 22 | 2 | 9.1 | Oncogene 2013;32:768–76 |
| Kalinsky | 590 | 21 | 3.6 | Clin Cancer Res 2009;15:5049–59 |
| Majewski | 355 | 0 | 0 | J Clin Oncol 2015;33:1334–9 |
| **Total** | **2990** | **93** | **3.1** |  |
